# Pseudo Double Bubble: Jejunal Duplication Mimicking Duodenal Atresia on Prenatal Ultrasound 

**Published:** 2013-10-01

**Authors:** David Schwartzberg, Sathyaprasad C Burjonrappa

**Affiliations:** 1 Monmouth Medical Center, USA; * University of Buffalo, USA

**Keywords:** Double bubble, Neonatal bowel obstruction, Duplication cyst

## Abstract

Prenatal ultrasound showing a double bubble is considered to be pathognomonic of duodenal atresia. We recently encountered an infant with prenatal findings suggestive of duodenal atresia with a normal karyotype who actually had a jejunal duplication cyst on exploration. A finding of an antenatal double bubble should lead to a thorough evaluation of the gastrointestinal tract and appropriate prenatal/neonatal testing and management as many cystic lesions within the abdomen can present with this prenatal finding.

## INTRODUCTION

Duodenal atresia has long been associated with the antenatal “double bubble” sign on ultrasound. This appearance results from a distended stomach and duodenal bulb that are separated by a hypoechoic gastric antrum. It is often associated with a collapsed small bowel and colon and maternal polyhydramnios. [1] While these findings are considered specific for obstruction at the level of the duodenum, specific signs such as a hyper-echogenic band in annular pancreas or anatomic location of the angle of Treitz in malrotation are not found in many instances to facilitate an exact diagnosis. [2] The “double bubble” monicker is mainly associated with duodenal atresia; however differential diagnoses in the literature clearly describe intestinal malrotation, midgut volvulus, annular pancreas and other right upper quadrant cystic lesions consistently. [1] We recently treated a neonate with an antenatal “double bubble” who on further evaluation was found to have a jejunal duplication cyst. The finding of an antenatal “double bubble” should include a thorough work up of the gastro intestinal (GI) tract beyond the ligament of Treitz as the causative pathology may not be restricted to the peri-ampullary area. 

## CASE REPORT

A full-term 2610g male neonate was admitted to the intensive care unit (NICU) with an antenatal history significant for a “double bubble” detected during the second trimester ultra sound examination (Fig. 1). A presumed diagnosis of duodenal stenosis/atresia was made with further karyotyping and anomaly scan showing no abnormalities. The antenatal imaging performed in the third trimester re-confirmed the findings of a double-bubble with no other anomalies being identified. Born at 38 +2/7 weeks, with APGAR scores of 7 and 9, the neonate had mild fullness of the right upper quadrant on clinical exam but no palpable mass. A supine abdominal x-ray revealed a dilated small bowel loop in the right upper quadrant with distal bowel gas, which was interpreted as a possible mega duodenum due to obstruction at that site consistent with the antenatal “double bubble” finding (Fig. 2). Patient then underwent an upper gastrointestinal series (UGI), which initially revealed no evidence of gastric outlet and duodenal obstruction or malrotation. However, subsequent delayed films showed of a space-occupying lesion in the right upper quadrant with a characteristic displacement of small bowel loops. Its subsequent opacification on delayed films was suggestive of a duplication cyst (Fig. 3). No evidence of bowel obstruction was seen. A post-natal ultrasound was not considered necessary in view of the findings on the UGI series.

**Figure:**
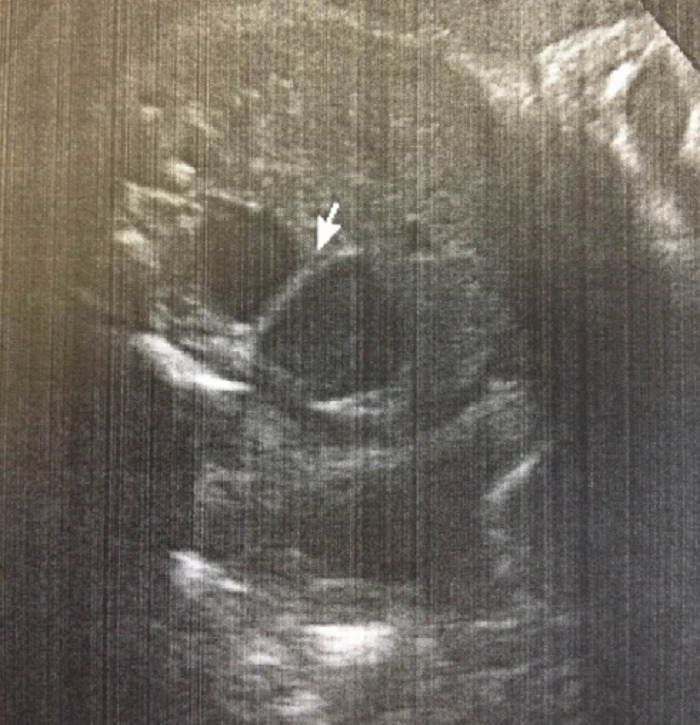
Figure 1: 22wk prenatal US showing pseudo double bubble.

**Figure:**
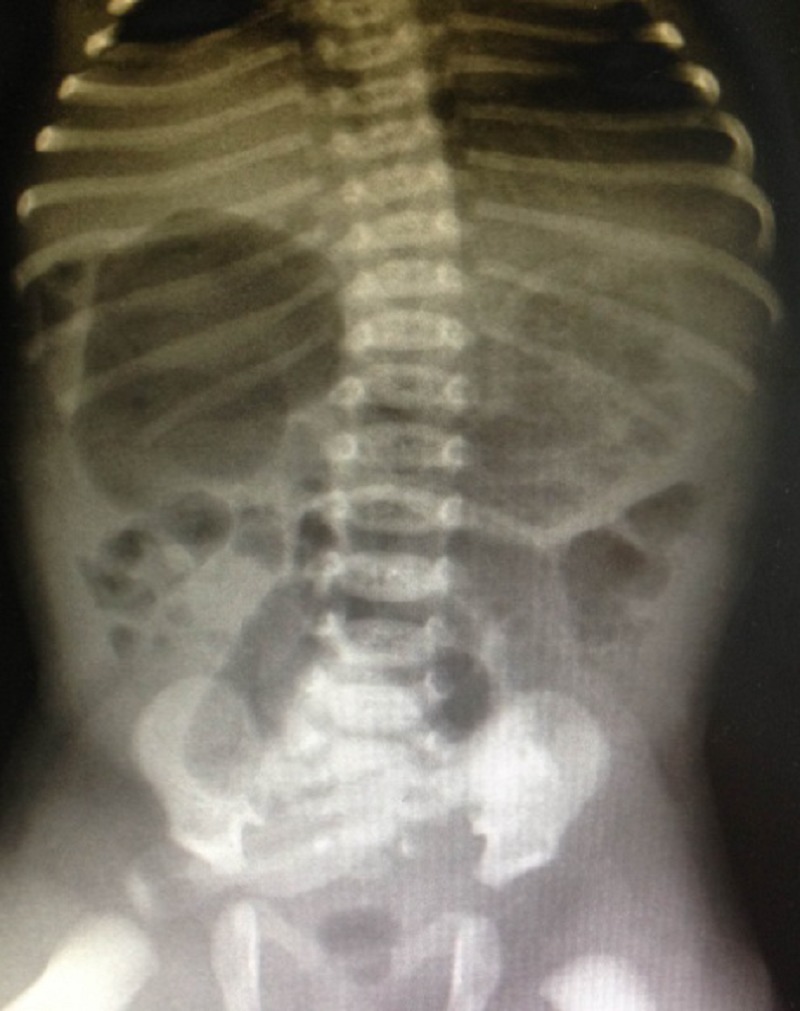
Figure 2: Neonatal flat plate, large gas bubble in RUQ suggestive of partial duodenal obstruction

**Figure:**
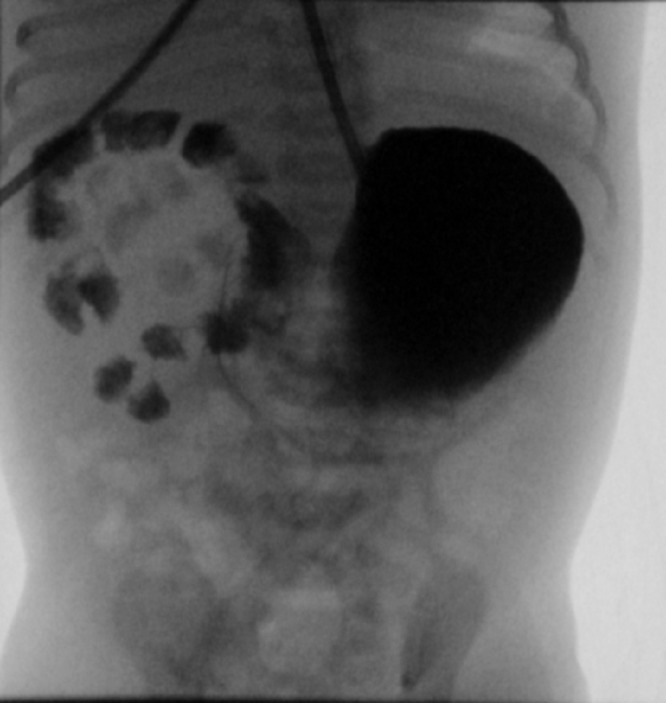
Figure 3: Delayed UGI films showing a duplication cyst of the jejunum

On day of life three, the patient underwent a diagnostic laparoscopy. Intra-operative findings included a 4x3 cm cystic mass in the right upper quadrant consistent with a duplication cyst (Fig. 4), as well as a freely mobile cecum in the left abdomen consistent with malrotation. A directed laparotomy (guided by the initial laparoscopic evaluation) with resection of duplication cyst and anastomosis of the small bowel along with a Ladd’s procedure were performed. No classical Ladd’s bands could be identified although there were numerous abnormal mesenteric bands compressing the distal small bowel that were divided. The patient was discharged home uneventfully after a two-week stay in the neonatal intensive care unit. 

**Figure:**
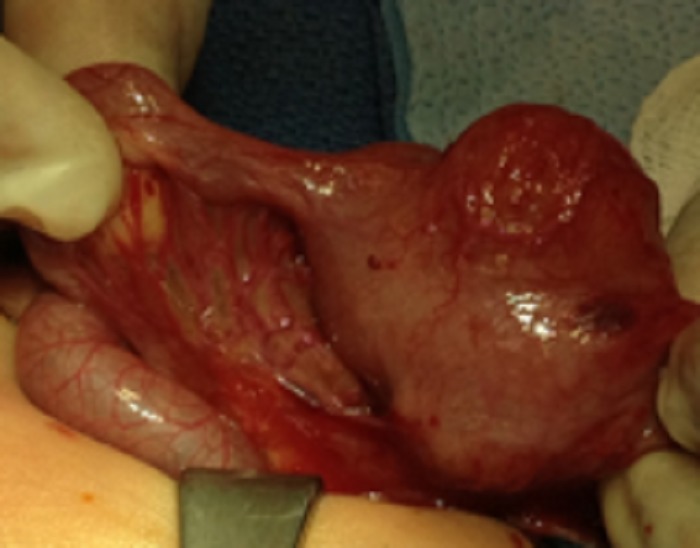
Figure 4: Operative findings of jejunal duplication cyst

## DISCUSSION

From the onset of the prenatal diagnosis of a “double bubble”, which is mainly a second trimester finding, exclusion of Down’s syndrome becomes an integral part of the management. An antenatal karyotype or a triple test (AFP, HCG and Estriol) must be completed to rule out Down’s syndrome. If, as has been rarely reported, the double bubble is detected in the first trimester, it may help facilitate further management of the pregnancy including counseling of the family with regards to their options including termination of pregnancy. The finding of a “double bubble” is non-specific and all differential diagnoses must be ruled out prior to the diagnosis of isolated duodenal atresia. Therefore additional antenatal ultrasonography should be performed to better visualize potential causes as well as neonatal tests to rule out conditions that require urgent operative intervention.[2, 3]

Starting at fourteen to fifteen weeks’ gestation, antenatal ultrasound can be used to visualize intra-abdominal organs. While accurate diagnosis of obstructions or abnormalities occur closer to twenty-two weeks gestation, at fourteen weeks a distended fluid-filled stomach appears in the left upper quadrant along with a normally under-distended small bowel. Small bowel distention is therefore indicative of distal GI pathology, either distal small bowel or colonic obstruction. [4] 

Duplication cysts are rare congenital malformations with an incidence of one in forty-five hundred live births. [3] These cysts always require resection either urgently because of obstructive symptoms or electively for potential malignant degeneration. Fifteen percent of the time there are synchronous cyst along the gastrointestinal tract; reports have documented occurrences from esophagus to rectum. [5] Duplication cysts are more common in males and have a predilection to the ileum. [3] Duplication cysts also have ultrasonographic criteria however they are not present in all occurrences. [7,9] Studies show that duplication cysts detected on antenatal ultrasound must include a double wall rim consisting of an inner hyperechoic rim of mucosa-submucosa, and an outer encompassing hypoechoic layer of muscularis propria. [8] Contrast radiography may show a mass lesion that communicates with the GI tract or an extra-intestinal mass displacing adjacent bowel loops.

The classic “double bubble” sign represents an over-distended stomach with an abnormally distended duodenal bulb. On transverse plane of an ultrasound this appears as two large echogenic lumens separated by a hypoechoic gastric antrum. [4] No dimensional requirements or criteria for the size of the lumens or the antrum exist as the non-specific finding of a double bubble is visualized with antenatal ultrasound. With that being said, ultrasonographic findings of a polyhydramnios (of variable severity) and double bubble are still historically pathognomonic for duodenal atresia, which occurs in one in every ten thousand live births. [4] The majority of duodenal atresias are isolated developmental vascular accidents, however thirty percent of cases are associated with trisomy 21 Down’s syndrome. [6] Other anomalies responsible for the visualization of an antenatal double bubble include, malrotation, volvulus, annular pancreas, duplication cysts, internal hernia, and cystic lesions of the right upper abdomen such as a choledochal cyst. [2,4] An UGI study or other postnatal imaging can be obtained to confirm the exact diagnosis. 

A double bubble detected on antenatal ultrasound is a serious finding that usually implies an obstruction proximal to the ligament of Treitz. It is well documented that there are many anomalies that mimic a double bubble sign so additional testing is mandatory. We herein report the first case of a large jejunal duplication cyst mimicking a double bubble sign: a “pseudo double bubble”. We believe based on our recent experience that in some neonates the causative pathology of an antenatal “double bubble” may lie far distal to the ligament of Treitz and a thorough evaluation of the entire GI tract is necessary to come to a correct diagnosis and perform the appropriate intervention. 

## Footnotes

**Source of Support:** Nil

**Conflict of Interest:** None

